# Pethidine, Maybe a Rearrangement in the Pharmaceutical Group Is Needed!

**DOI:** 10.5812/ijpr-156667

**Published:** 2024-12-06

**Authors:** Reza Aminnejad, Ali Solhpour, Sahar Kavousi Sisi

**Affiliations:** 1 Shahid Beheshti Hospital, Qom University of Medical Sciences, Qom, Iran; 2 Clinical Research and Development Center (CRDC), Qom University of Medical Sciences, Qom, Iran; 3 Department of Anesthesiology, University of Florida, Gainesville, USA; 4 Department of Clinical Pharmacy, School of Pharmacy, Shahid Beheshti University of Medical Sciences, Tehran, Iran

**Keywords:** Pethidine, Meperidine, Shivering, Postoperative Pain

*Dear Editor,*

Pethidine (meperidine) was discovered in 1939 and has long been recognized as an analgesic in routine clinical practice (1). Painless delivery is one of the oldest fields in which this drug has been used for analgesia (2). In recent years, many concerns have been raised regarding the use of pethidine as an analgesic (3). Seizures are a major concern with meperidine use, as it is one of the drugs with high epileptogenic potential (4). Normeperidine, a neurotoxic metabolite of meperidine, is known to be the primary culprit in causing seizures. Although the risks of pethidine use in patients with liver dysfunction are well established, long-term use, especially at high doses, can also be hazardous for individuals without hepatic impairment. The key factor is the individual's cytochrome system, which determines the production of this dangerous metabolite (5). 

Seizures are not the only risks associated with pethidine. Meperidine is considered the most dangerous opioid for causing perioperative delirium (6, 7). However, some authors argue that concerns about the use of this drug are exaggerated (8). In response to this perspective, two points should be considered. First, when equally or more effective drugs are available and have been proven safer, what justifies the use of an older medication with documented safety concerns? Secondly, even these authors do not provide definitive recommendations, leaving final conclusions to further future research.

The reality is that pethidine use has declined over the past two decades due to growing concerns regarding its adverse effects, primarily seizures and serious drug-drug interactions (9). However, its relatively exclusive property as an antishivering agent remains noteworthy. Although no definitive gold standard for the prevention and treatment of shivering has been established, pethidine consistently appears at the forefront of any list of effective medications for this purpose (10-13). 

Given that the most rational use of pethidine today is for the prevention and treatment of shivering, it may be more appropriate to refer to this drug as an antishivering agent rather than a narcotic analgesic. This reclassification could help shift the traditional perspective of pethidine as a primary analgesic for surgical patients, encouraging the use of newer and safer drugs for pain management while reserving pethidine for its unique antishivering properties.
